# Supplementary material for: Eco-Friendly Activation of Silicone Surfaces and Antimicrobial Coating with Chitosan Biopolymer
Source: Int J Mol Sci. 2025 Dec 16;26(24):12084. doi: 10.3390/ijms262412084 (PMC12733018; doi:10.3390/ijms262412084)
Supplement: Supplementary file 1 [file ijms-26-12084-s001.zip › ijms-3980438-supplementary.pdf]

Supporting Information

Eco-Friendly Activation of Silicone Surfaces and Antimicrobial Coating with Chitosan Biopolymer

Daniel Amani [1], Guðný E. Baldvinsdóttir [1], Vivien Nagy [2], Freygardur Thorsteinsson [3] and Már Másson [1,*]

1 Faculty of Pharmaceutical Sciences, School of Health Sciences, University of Iceland, Hagi, Hofsvallagata 53, 107 Reykjavík, Iceland

2 Minamo, Hofsvallagata 53, 107 Reykjavík, Iceland

3 Össur Iceland ehf., Grjóthálsi 5, 110 Reykjavík, Iceland

* Correspondence: mmasson@hi.is

1. Supporting Experimental Details

1.1. Contact angle analysis of modified silicone surfaces

Static water contact angles were measured using an optical tensiometer in sessile drop mode. Silicone, ETA-activated silicone and chitosan-coated silicone samples were placed on a leveled stage and equilibrated at room temperature (≈20 °C). A 5 µL droplet of deionized water was dispensed onto the surface using an automated syringe, and images were recorded for 90 s with a time interval of 0.12 s between consecutive frames (0–90 s). For each frame, the left and right contact angles were determined by Young–Laplace fitting, and the mean contact angle (CA[M]) was calculated by the instrument software. The experiment was performed with three replicates for each sample, and the average CA[M] values were used to construct the contact angle–time curves.

1.2. Scanning electron microscopy of silicone surfaces

Scanning electron microscopy (SEM) was used to examine the surface morphology of untreated silicone, ETA-activated silicone, and chitosan-coated silicone. Disc-shaped samples with a diameter of 12 mm were mounted on aluminum stubs using carbon tape and sputter-coated with a thin layer of gold to minimize charging. SEM images were acquired using a operated at an accelerating voltage of 10 kV and a working distance of ~6.9 mm. Each surface was imaged at low and high magnifications (scale bars 10 and 100 µm) to assess overall morphology and fine surface features.

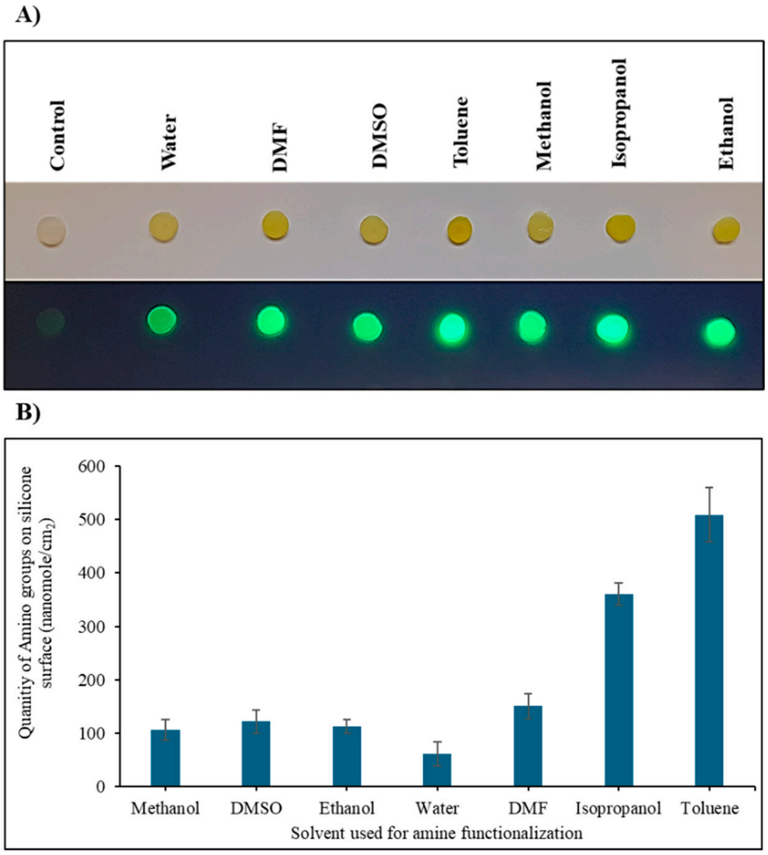

Figure S1. (A) Image of unactivated silicone disc (control) and ETA-activated silicone discs treated with FITC in different reaction media under normal light and UV light. (B) Quantification of amino groups on the silicone surface using 10% ETA in different reaction media. The values represent the mean ± standard deviation.

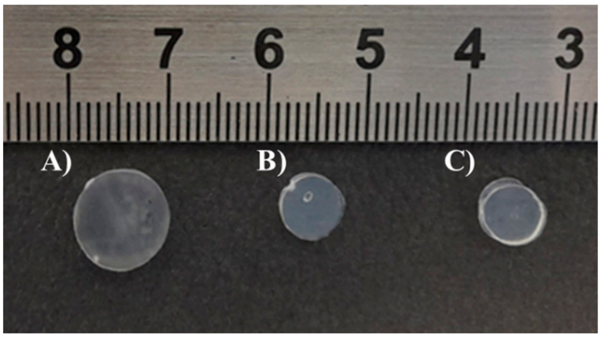

Figure S2. Representative image of silicone discs: (A) after treatment with 10% ethanolamine in toluene, (B) after subsequent washing with isopropanol and water, and (C) untreated silicone control.

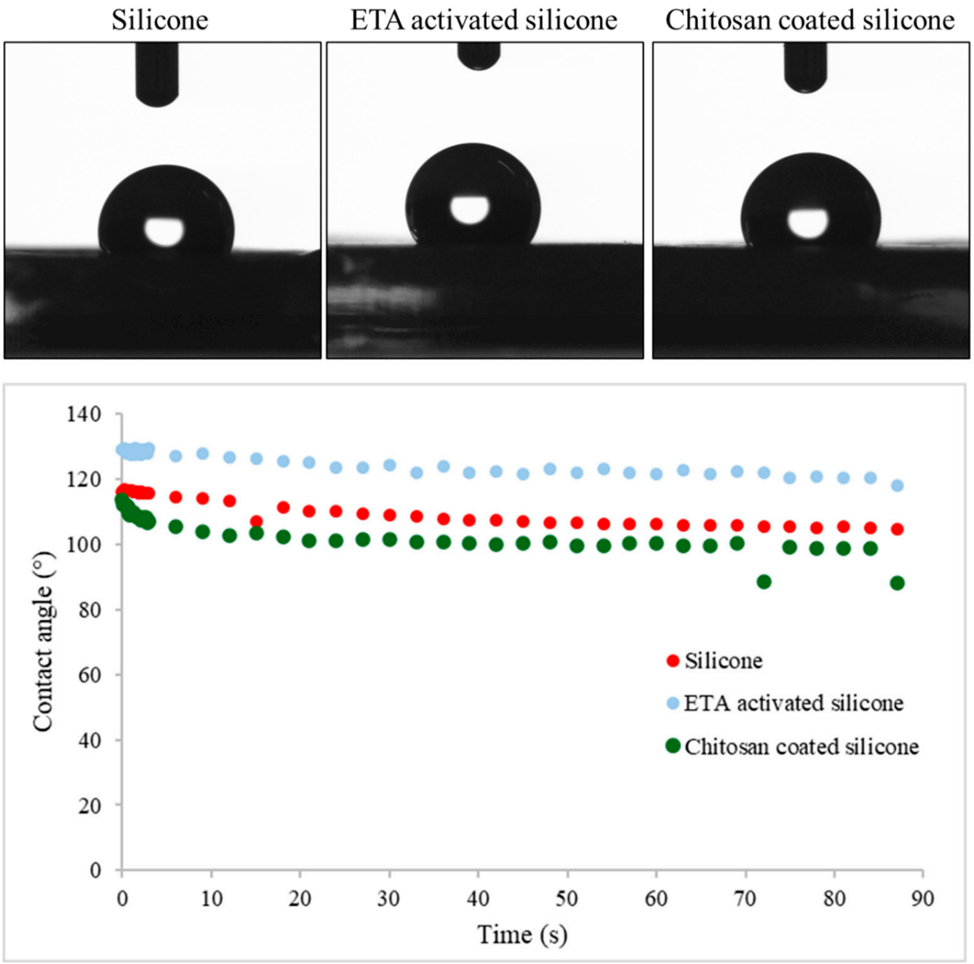

Figure S3. Water contact angle images and dynamic contact angle measurements over 90 s for untreated silicone, ETA-activated silicone, and chitosan-coated silicone.

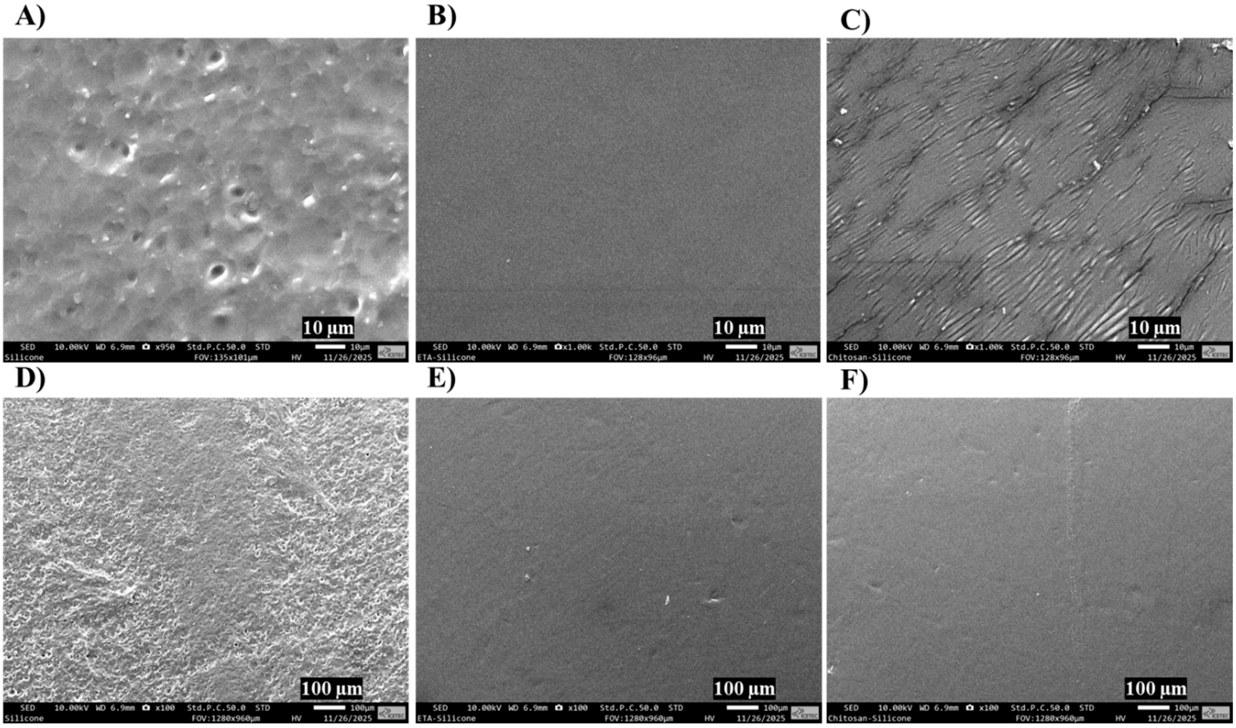

Figure S4. Scanning electron microscopy (SEM) images of silicone surfaces: (A, D) untreated silicone; (B, E) ETA-activated silicone; (C, F) chitosan-coated silicone.

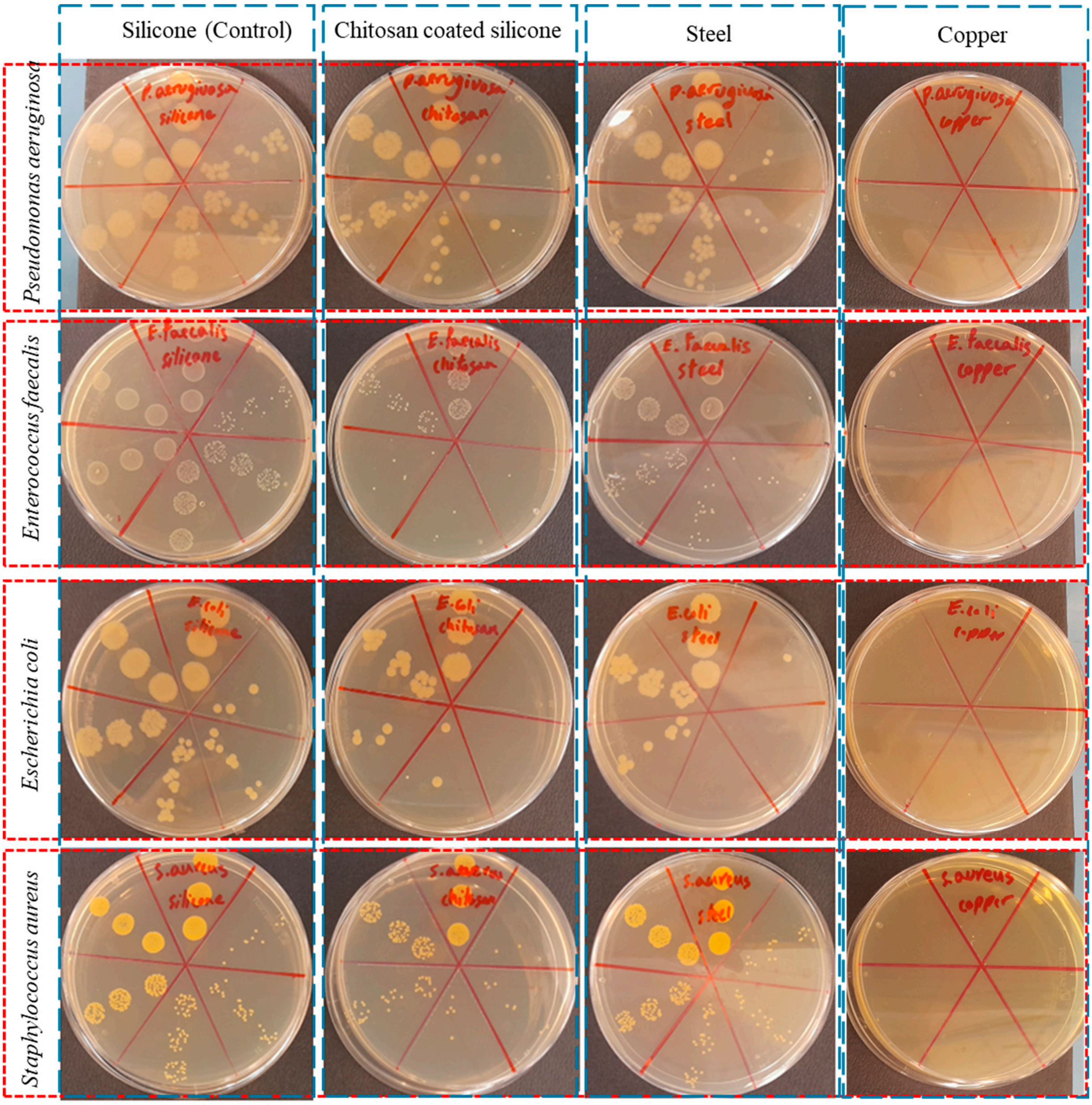

Figure S5. Antibacterial efficacy of silicone, chitosan coated silicone, steel and copper discs against various bacterial strains.
